# Anti-Obesity Action of *Boerhavia diffusa* in Rats against High-Fat Diet-Induced Obesity by Blocking the Cannabinoid Receptors

**DOI:** 10.3390/plants11091158

**Published:** 2022-04-25

**Authors:** Mohammad Khalid, Mohammed H. Alqarni, Ambreen Shoaib, Shadma Wahab, Ahmed I. Foudah, Tariq M. Aljarba, Juber Akhtar, Mubarak A. Alamri, Sarfaraz Ahmad

**Affiliations:** 1 Department of Pharmacognosy, College of Pharmacy, Prince Sattam Bin Abdulaziz University, P.O. Box 173, Al-Kharj 11942, Saudi Arabia; m.alqarni@psau.edu.sa (M.H.A.); a.foudah@psau.edu.sa (A.I.F.); t.aljarba@psau.edu.sa (T.M.A.); 2 Department of Clinical Pharmacy, College of Pharmacy, Jazan University, P.O. Box 114, Jazan 45142, Saudi Arabia; asahmad@jazanu.edu.sa (A.S.); sarfaraz3030@gmail.com (S.A.); 3 Department of Pharmacognosy, College of Pharmacy, King Khalid University, P.O. Box 960, Abha 61421, Saudi Arabia; shad.nnp@gmail.com; 4 Department of Pharmaceutics, Faculty of Pharmacy, Integral University, Lucknow 226026, India; juberakhtar@gmail.com; 5 Department of Pharmaceutical Chemistry, College of Pharmacy, Prince Sattam Bin Abdulaziz University, P.O. Box 173, Al-Kharj 11942, Saudi Arabia; mubarak@psau.edu.sa

**Keywords:** *Boerhavia diffusa*, boeravinone B, punarnavine, Eupalitin, cannabinoid receptors, obesity

## Abstract

Obesity, type 2 diabetes, and cardiovascular illnesses have known risk factors in the pathophysiology of an unhealthy diet. Obesity now affects almost a third of the world’s population and is widely seen as a side effect of the Industrial Revolution. The current study aimed to determine natural phytoconstituents that have a significant role in the management of obesity. In this view, we have selected the plant *Boerhavia diffusa* which has different pharmacological actions and is traditionally used to treat sickness caused by lifestyle modification. The methanolic extract of the plant material was prepared and then further fractionated by means of solvents (n-hexane, chloroform, n-butanol, and water). The absorption, distribution, metabolism, excretion, and toxicity (ADMET) analysis was done by taking the active constituent of the plant (Punarnavine, Boeravinone B, and Eupalitin). The molecular docking analysis of these compounds is also performed by targeting the cannabinoid receptor (CR). Structural analysis of the best complex was done using the Discovery Studio visualizer tool. High-performance thin-layer chromatography (HPTLC) analysis was done by using a solvent system (chloroform and methanol in a ratio of 8:2). The *in vivo* study was done on the Sprague–Dawley (SD) rats treated with a high-fat diet to induce obesity and different parameters such as body weight, behavioral activity, organ fat pad weight, lipid profile, and liver biomarkers (AST, ALT, BUN, and creatinine) were estimated. The result of the study suggested that the phytoconstituents of *B. diffusa* upon molecular docking revealed the possible binding mechanisms with the CR and thus show potent anti-obesity action.

## 1. Introduction

Ayurveda takes part in the Indian civilization and serves in managing obesity, cardiovascular disorders, liver disorder, and diuretics to control and treat various oedematous conditions associated with health problems [1]. Ayurveda plays an important role in obesity as it is ranked a global health issue by the World Health Organization (WHO) [2]. According to WHO, depending on the Body Mass Index (BMI), the human being is classified into standard (18.5–24.9 kg/m^2^), over-weight (24.9–29.9 kg/m^2^), and severely obese (more than 30 kg/m^2^). Over a few decades, obesity has drastically increased and has become a global health issue. Due to the sedentary lifestyle of the younger generation, virtually more than 850 million individuals were considered obese across the globe. Thus, there is an unmet need for the management of obesity and that can only happen through strict regulation of diet and working out. Several medications are used for this purpose and they are termed anti-obesity drugs. Some of them are approved by the European Medicines Agency along with the United States Food and Drug Administration [3].

Obesity has been related to environmental and genetic variables that predispose people to the disease. Obesity, a global pandemic on the rise, is not being adequately managed by existing lifestyle changes, bariatric surgery, or accessible drugs. The toll it takes on people’s health and the economy forces us to take more effective measures [4]. Moreover, the patients are not skilled in learning good eating behavior, and they generally regain weight after treatment. The highest dose recommended of any herbal preparations containing phytoconstituents will not contribute to these problems, although it cannot disturb the basal metabolism [5]. Flavonoids and phenolic compounds are abundantly distributed in fruits, seeds, and vegetables [6]. Currently, phenolic and flavonoids compounds have potential anti-obesity activities [7].

Calorie intake, thermodynamic efficiency, and body mass are all influenced by the cannabinoid system [8]. Ethnopharmacological treatment is now required for obesity. Cognition and appetite are two of the many targets investigated in this approach. The cannabinoid system, with endogenous ligands anandamide, 2-arachidonoyl glycerol (2-AG), regulates food intake as well as a wide range of pharmacological activities. The active constituent of marijuana, 9-tetrahydrocannabinol, is shown to cause bulimia in humans [9,10]. When given to rats, the endogenous cannabinoids anandamide and 2-AG promote eating [11].

*Boerhavia diffusa* commonly known as Spreading Hogweed is a perennial herbaceous creeper weed. In Indian tradition, it is recognized by the name Punarnava [12]. Punarnava root contains punarnavine, xanthine derivatives, ursolic acid, and β-sitosterol. 5-dihydroxy-3, 4 dimethyl flavone glucose, and hypoxanthine-9-arabinoside. It also contains purine nucleoside hypoxanthine-9-arabinofuranoside and rotenoid analogs like boeravinones A–F [13]. Apart from all these constituents, inorganic salts such as potassium nitrate, potassium sulfate, and chloride are also present [14].

The medicine is used for eye diseases in Punjab and dropsical swellings in Bombay. The root is used as a laxative, and also for the management of inflammation and urinary illness [15]. The plant’s methanolic extracts were efficient in suppressing metastases in some melanoma cells [16]. It is also a powerful antidote for snake and rat bites [17]. Punarnava helps cure nephrotic syndrome [18]. Mudgal reported punarnava root’s benefits for hepatitis, gall bladder issues, and urinary issues in 1975. The seeds and petals are contraceptives [19]. Many researchers claim that plants are rich in vitamins, minerals, protein, and carbs, as well as alkaloids, flavonoids, saponins, and steroids [20,21]. Almost all studies focus on root and leaf chemistry [22]. Pari and Amernath (2004) found that the leaves of *B. diffusa* lowered blood glucose levels via increasing insulin release from pancreatic cells [23].

Therefore, the present study utilized herbal medicine derived from the plant source (*B. diffusa*). Due to the importance of standardization, we worked on the HPTLC profile of the plant extract. To elucidate the underlying mechanism of suppression of obesity, the absorption, distribution, metabolism, excretion, and toxicity (ADMET) analysis and molecular docking were performed. To prove its anti-obesity action, the *in vivo* activity was evaluated.

## 2. Materials and Methods

### 2.1. Chemicals/Reagents

#### 2.1.1. Extracts Preparation

It took three weeks to dry the roots to a steady weight. The dried roots were then powdered, with 500 g of raw powdered plant material orbitally shaken in methanol for 72 h at room temperature, with handshaking every 24 h. The extract was filtered and dried at 40 °C by means of an evaporator under reduced pressure [24].

#### 2.1.2. Fractionations of Methanolic Extracts

Using a separating funnel, the methanolic extract was separated into fractions by using n-hexane, chloroform, n-butanol, and water. On a rotary evaporator under reduced pressure, all fractions were concentrated to dryness, yielding 2, 3.5, 4.2, and 5.75% yield in hexane, chloroform n-butanol, and water correspondingly [21,25].

### 2.2. ADMET Analysis Punarnavine, Boeravinone B, and Eupalitin

The drug-like properties of Punarnavine, boeravinone B, and Eupalitin were determined following Lipinski’s rule of 5 using the online Molinspiration database (www.molinspiration.com accessed on 5 March 2022) and AdmetSAR. The parameters that were considered were molecular weight (MW), lipophilicity (log P), N atoms, GPCR score, nuclear receptor ligand score, Topological surface area (TPSA), and human oral absorption [26].

### 2.3. Molecular Docking

#### 2.3.1. Target Preparation, Ligand Retrieval, and Energy Minimization

The structure of the Cannabinoid receptor (PDB ID: 5TGZ) was cleaned by deleting the heteroatoms and the crystallographically observed water molecules (water lacking H-bonds), correcting errors in the PDB file. The ligands’ structure was drawn via ChemDraw (Version 12.0.2, licensed: Mulder 2010 Cambridge soft) to obtain the mol file and converted into PDB by Discovery studio visualizer. Energy minimization of Cannabinoid Receptor (CR) was done using Swiss-PDB Viewer [27].

#### 2.3.2. Molecular Docking Analysis

Selected compounds (Punarnavine, boeravinone B, and Eupalitin) were docked at the active site residues of CR by engendering the PDBQT, Grid parameter file (GPF), and a Docking parameter file (DPF) using Autodock 4.2 software (The Apache Software Foundation, 1000 N West Street, Suite 1200 Wilmington, DE 19801, USA). These files were run on a Cygwin terminal to get the output files, namely the Grid log file (GLG) and a Docking log file (DLG). The complexes with the lowest binding energies were chosen for the best conformation. Structural analysis of the best complex was done using the Discovery Studio visualizer tool [27].

### 2.4. GROMACS 2018 Molecular Dynamics Simulation Studies

The OPLS-AA all-atom force field and the TIP3P water model were used to run the simulation studies for the best-docked poses of the reference protein, sibutramine, punarnavine, boeravinone B, and eupalitin complexes with ductal cannabinoid receptor (PDB ID: 3TGZ). The SwissParam online service (https://www.swissparam.ch/ (accessed on 10 February 2022)) generates topographical parameters for simulated molecules Khalid et al. The complexes were solvated and neutralized in a cubic box separated by at least 1 nm. The counter ions employed were 0.15 M (Na + Cl). The complexes’ energy usage was subsequently reduced using the steepest descent technique. The lenience was 1000 kJ/mol/nm with a 0.01 nm step size. Bond length restrictions were made with the Linear Constraint Solver (LINCS; Parallelization of the algorithm is straightforward. © 2022 John Wiley & Sons, Inc.). Electrostatic calculations were carried out using the PME method. Equilibration was performed for 100 ps using the NVT and NPT (isobaric–isomeric ensembles). A 20-ns production run ended after Plants 2022, 11, 562 6 of 19. Key MD parameters including RMSD, RMSF, HB, and Rg were studied using GROMACS 2018.1 packages.

#### 2.4.1. HPTLC Analysis of Extracts

The methanolic extract was utilized to estimate TLC using CAMAG HPTLC (win CATS Software Version 1.4.4.). Assembly with TLC Scanner 3 and with the CATS program. We utilized 10 × 10 cm HPTLC plates silica gel 60 F254 for this experiment (E. Merck KGaA, Darmstadt, Germany). The plates were activated for an hour at 60 °C in a hot air oven. The solvent system was impregnated into a clean dry Twin-trough chamber (20 × 10 cm) (chloroform and methanol in a ratio of 8:2). The chamber was closed to let the solvent fumes fill it. The samples were spotted on the stimulated HPTLC plate using the LINOMAT V under inert gas pressure. The stained plates were stored in chromatographic solvent chambers. Glass plates carefully covered the chambers. The plates were then removed from the solvent system and allowed to dry at ambient temperature. They were sprayed with freshly made vanillin sulfuric acid. The HPTLC system and TLC Scanner 3 evaluated the sprayed and air-dried plates [28]. The scanning chromatogram and spot Rf values were recorded.

#### 2.4.2. Animals

SD rats (120–140 gm) were procured from the National Laboratory Animal Centre, Central Drug Research Institute, Lucknow, India. The animals were housed separately in a polypropylene cage for one week before and during the experiment, with a 12-h light/dark cycle. Various experimental groups of animals had free access to high-fat meals (Composition Ingredients Quantity in g/kg: Casein 342.0, L-Cystine 3.0, Starch 172.0, Sucrose 172.0, Cellulose 50.0, Groundnut oil 25.0, Tallow 190.0, AIN salt mix 35.0, AIN vitamin mix 10.0, Total (g) 999.0) and water. All experimental measures followed the Committee’s Control and Supervision of Animal Experiments guideline (CPCSEA). The Integral University, Faculty of Pharmacy, Lucknow, India, Institutional Animal Ethics Committee authorized the study procedures (IU/Pharma/Ph.D./CPCSEA/10/18).

#### 2.4.3. Experimental Study

Group I was the normal control group and was treated with a normal diet, Group II was the positive control group and was treated with only a high-fat diet (HFD), Group III was served with HFD along with sibutramine (5 mg/kg b.w.), Group IV was treated with a HFD with n-hexane fraction (Hx-F) of the *B. diffusa* extract at a dose of 200 mg/kg b.w., whereas Group V was treated with a HFD along with chloroform fraction of *B. diffusa* (Chl-F) at a 200 mg/kg b.w. dose in the same way Group VI was treated with HFD plus n- Butanol fraction of *B. diffusa* (nBut-F) at a minimal dose of 200 mg/kg b.w., and, last, there was Group VII, the treatment group served a HFD with water fraction of *B. diffusa* (Wt-F) at 200 mg/kg b.w. All the groups were served via an oral route for a period of 60 days.

#### 2.4.4. Estimation of Parameters

##### Determination of Behavioral Activity and Body Weight

Each group was observed daily for the behavioral changes and every week the body weight (g) was recorded for each animal till the study ends [29].

##### Determination of Weights of Organs and Fat Pads

The rats were sacrificed by cervical dislocation, and multiple organs were removed and weighed to measure fat pad density, including the heart, kidney, liver, and uterus fat pads [29].

##### Determination of Blood Parameters

For the estimation of different parameters, we have anesthetized the animals by the means of diethyl ether. At the end of the experiment, a blood sample was taken from the tail vein, and the animals were sacrificed.

##### Effects of Wt-F of *B. diffusa* Extract on Lipid Profile

The serum level of total cholesterol, high-density lipoprotein, low-density lipoprotein, very low-density lipoprotein, and triglyceride was estimated by methods [30,31].

##### Effects of Wt-F of *B. diffusa* Extract on Liver Biomarkers (AST and ALT) Levels

The liver biomarker especially the AST and ALT were performed [32].

##### Effects of Wt-F of *B. diffusa* Extract on Blood Urea Nitrogen and Creatinine

Serum blood urea nitrogen (BUN) and creatine were estimated as per the method mentioned on commercially available span diagnostic kits [33,34].

#### 2.4.5. Statistical Analysis:

A mean SEM was used for all data. One-way analysis of variance was used to examine the statistical significance of various sets (ANOVA). This was followed by Dunnett’s test software graph pad (Version 3.0, 2003, Graph pad software Inc. 11462, San Digeo, CA, USA). (*p* < 0.05).

## 3. Results

### 3.1. HPTLC Analysis

**Figure 1:**
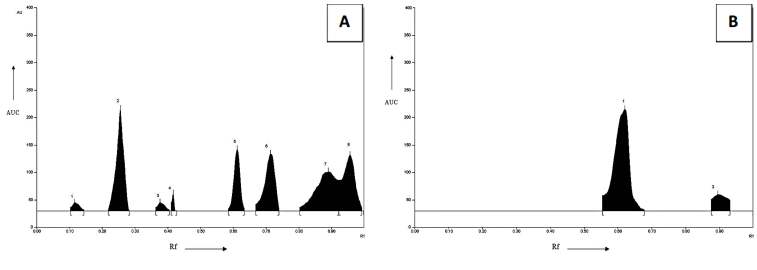
(**A**) HPTLC fingerprinting profile of *B. diffusa* extract. The peaks obtained after HPTLC fingerprinting of *B. diffusa* extract were similar to the peaks of phytoconstituents (Punarnavine and boeravinone B). Some additional peaks were also obtained; these may be a mixture of alkaloids. (**B**) HPTLC fingerprinting of *B. diffusa* extract. At 366 nm, the peaks obtained were nearly similar to the peaks of phytoconstituents studied before.

Table 1 is the explanation of the Rf values and the area of each spot of the HPTLC of fingerprints of methanolic extracts. Figure 1A is defining the HPTLC fingerprinting profile of *B. diffusa* extract. The peaks obtained after HPTLC fingerprinting of *B. diffusa* extract were similar to the peaks of phytoconstituents (Punarnavine and Boeravinone B). Some additional peaks were also obtained at 254nm, Rf values 0.11, 0.28,0.38, 0.43, 0.61, 0.71, 0.90, 0.96, and the area 625.52, 4821.79, 635.28, 498.76, 4338.61, 4487.26,4651.92, 4367.64 respectively that would be a mixture of alkaloids. Figure 1B is defining the HPTLC fingerprinting of *B. diffusa* extract. at 366 nm, the peaks obtained were nearly similar to the peaks of phytoconstituents of Rf values 0.61, 0.90, and the area obtained were 6647.29, 1029.34 respectively which were Punarnavine or Boeravinone B or both.

### 3.2. ADMET Predicted Profile and Pharmacokinetics Studies of Punarnavive, Boerhaavine B and Eupalitin

The ADMET properties of punarnavine, boeravinone B, and Eupalitin are shown in Table 2. Punarvavine was observed to have a smaller topological surface area (TPSA) in contrast to punarvavine and boeravinone B. The drug-likeness properties analyzed by PreADMET revealed that selected compounds have good human intestinal absorption (HIA). Distribution of Punarvavine, Boeravinone B, and Eupalitin was found to be in mitochondria. Selected compounds had shown Caco-2 cell permeability and the penetration strength of boeravinone B through the blood-brain barrier is weaker when compared to Eupalitin and Punarnavine. Eupalitin has shown a good ligand score against nuclear receptors and a higher log P (3.01) value than Punarvavine and boeravinone B. In addition, selected compounds qualified Lipinski’s RO5-like rules successfully and were predicted to be non-mutagenic and non-carcinogenic to mice and rats. The pharmacokinetics profile of the selected bioactive compounds (of Punarnavive, Boerhaavine B, and Eupalitin) are mentioned in Table 3.

### 3.3. Molecular Docking

**Figure 2:**
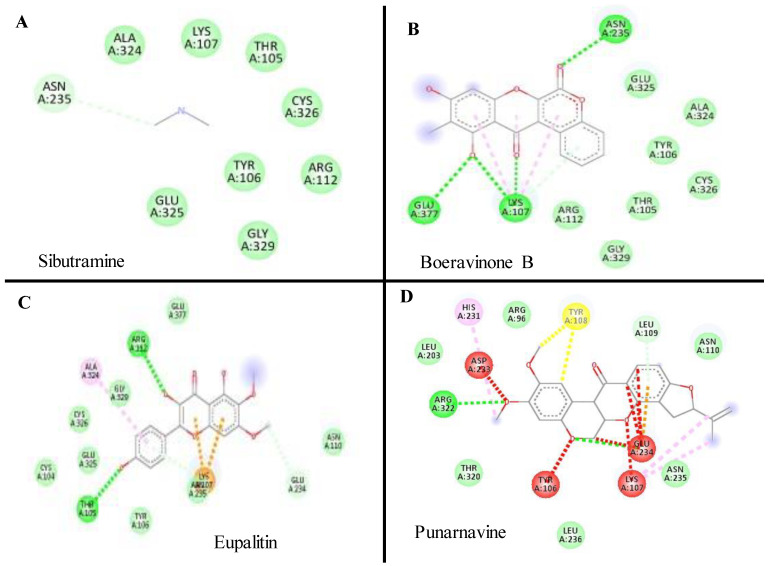
The docked complexes of selected compounds (**A**) Sibutramine, (**B**) Boeravinone B, (**C**) Eupalitin, and (**D**) Punarvavine with Cannabinoid receptor.

**Figure 3:**
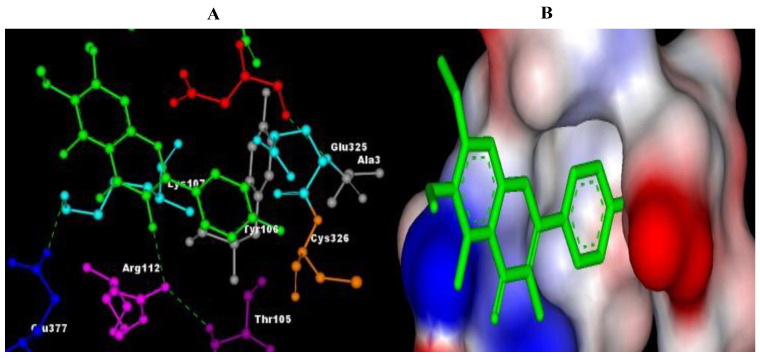
Pictorial representation of molecular docking analysis. (**A**) Binding orientation in the docked complex of CR with Eupalitin. (**B**) Surface representation of the CR binding pocket with the Eupalitin. The compound Eupalitin is represented in green. Images were generated using Discovery Studio visualizer.

**Figure 4:**
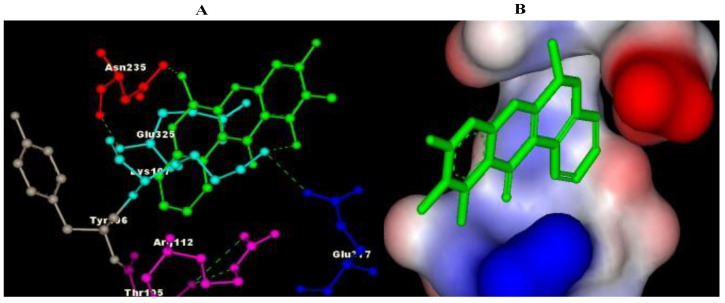
Pictorial representation of molecular docking analysis. (A) Binding orientation in the docked complex of CR with BB. (B) Surface representation of the CR binding pocket with the BB. The compound BB is represented in green. Images were generated using Discovery Studio visualizer.

**Figure 5:**
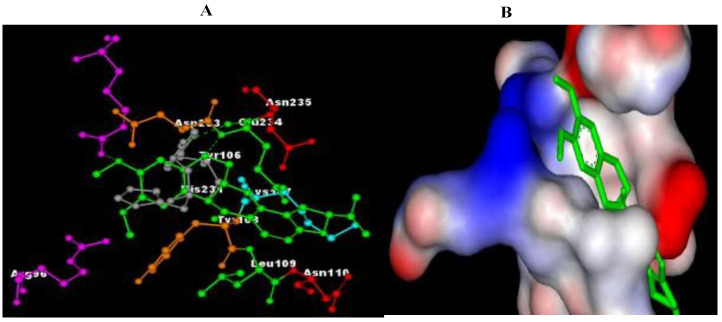
Pictorial representation of molecular docking analysis. (**A**) Binding orientation in the docked complex of CR with Punarnavine. (**B**) Surface representation of the CR binding pocket with the Punarnavine. The compound Punarnavine is represented in green. Images were generated using Discovery Studio visualizer.

**Figure 6:**
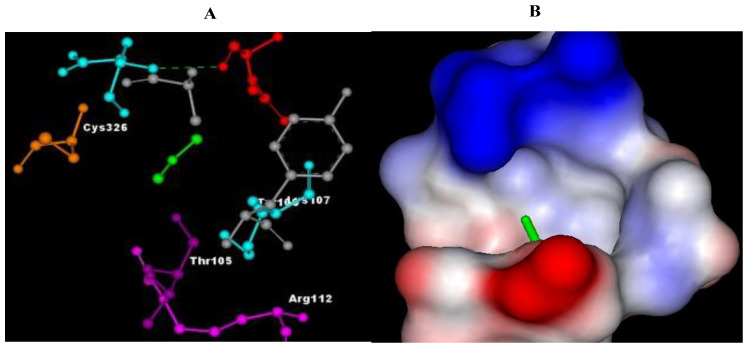
Pictorial representation of molecular docking analysis. (**A**) Binding orientation in the docked complex of CR with sibutramine. (**B**) is represented in green. Images were generated using Discovery Studio visualizer. Surface representation of the CR binding pocket with the sibutramine.

The results of molecular docking revealed the possible binding mechanisms of Cannabinoid receptors with the selected compounds. The binding energies (ΔG) of the selected compounds with the Cannabinoid receptor, the amino acid residues of the active site involved in interactions, the H-bond atoms involved, and the H-bond distance are mentioned in Table 4. Negative binding energy analysis showed a relatively strong affinity of the different ligands with the targeted protein. After evaluating the molecular docking analysis, Eupalitin has shown solid binding affinity with CR (−4.28 Kcal/mol), whereas Sibutramine has demonstrated a poor binding relationship with CR (+61 Kcal/mol). The images of the docked complexes of selected compounds with CR are shown in Figure 2, Figure 3, Figure 4, Figure 5 and Figure 6.

### 3.4. Molecular Dynamics (MD) Simulations

### 3.4.1. Total Potential Energy Calculations

**Figure 7:**
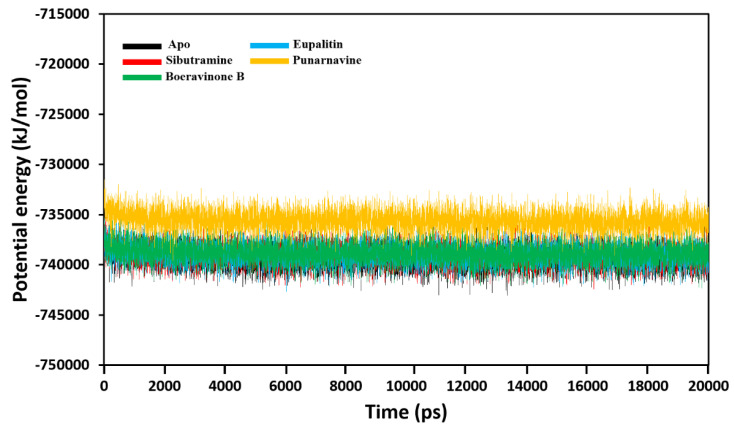
Total potential energies (kJ/mol) of the apo CB1 protein and the CB1–ligand complexes during the total 20 ns simulation time.

Molecular dynamics simulations were used to evaluate the dynamic stability of ligand complexes with CB1 for the CB1 in complex with sibutramine as a standard compound. The total potential energies of the apo CB1 receptor and in complex with sibutramine, eupalitin, boeravinone B, and punarnavine were computed for 20 ns. It was found that the energy minimization of the initial structure conformations at 0 ps was −739,324.0375, −739,050.4027, −738,899.6695, −738,841.3053, and −735,574.4577 kJ/mol, respectively. Throughout the 20 ns simulation period, the total potential energies of all systems were steady (Figure 7). All CB1 complexes showed a similar potential energy pattern as same as the apoprotein. The CB1-punarnavine complexes had the highest potential energy values than other systems. All complexes were stable and inconsistent with the docking study.

### 3.4.2. Stability Analysis

**Figure 8:**
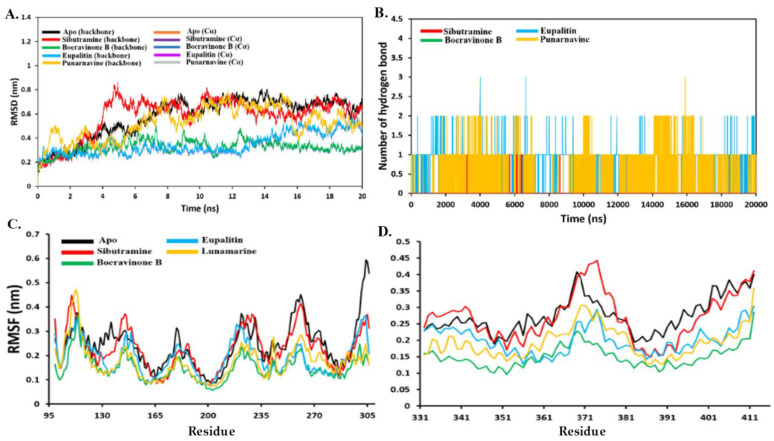
(**A**) The RMSD of the whole backbone atoms and alpha-carbon atoms (Cα) and for apo CB1 receptor and CB1–ligand complexes. (**B**) Several hydrogen bonds formed during the period of 20 ns MD simulation. (**C**) Residue flexibility analysis by RMSF per residue with a focus on 101–300 and in figure (**D**) at 333–410 residues.

To compare the structural stability of CB1 and the docked CB1 complexes, the atomic root means square deviations (RMSDs) of backbone and Cα atoms in CB1 and the docked CB1 complexes were evaluated (Figure 8A). All systems had reached equilibrium after 8 ns. Among all complexes, the boeravinone-B and eupalitin-CB1 complexes had lower RMSD values. Indicating that, both complexes were more stable than the other complex in terms of RMSD. Additionally, the binding of these compounds may help reduce the protein-energy and provide more stability to the protein. The sibutramine-CB1 complex showed high fluctuation between 4 to 6 ns then stabilized. Punarnavine-complex behaves as same as the apoprotein. In terms of local residues mobility, all systems showed low RMSF values in comparison to the apoprotein including the control complex; sibutramine-CB1(Figure 8B). This supports the RMSD results that the binding of these ligands to the CB1 receptor made the protein stable and less flexible. To confirm the docking study and provide more analysis on the driving forces for the binding of these ligands to the CB1, the number of hydrogen bonds formed during the MD simulation period was evaluated (Figure 8C). Inconsistent with the docking analysis, the eupalitin was able to form up to 3 hydrogen bonds with CB1 followed by other ligands which were able to interact with the CB1 receptor with 2 hydrogen bonds.

### 3.5. Pharmacological Studies

#### 3.5.1. Effect of Wt-F of *B. diffusa* Extract on Body Weight in SD Rats

##### Bodyweight

The HFD-treated group animals’ final body weight was 320.00 ± 5.01 g, which is substantially higher (*p* < 0.01) than the NC animals in-group II. The bodyweight of group III animals was 226.67 ± 2.01 g, which was substantially lower (*p* < 0.01) than group II animals. When group VII animals were compared to group II animals, they had a substantial decrease in body weight (228.73 ± 4.17, *p* < 0.01). In comparison to group II animals, the end bodyweight of group IV, V, and VI animals revealed no significant differences. Due to metabolic and 5HT pathways. Administration of Wt-F at a dose of 200 mg/kg/day was able to significantly reduce the body weight (*p* < 0.01) whereas the rest of the fractions (Hx-F, Chl-F, nBut-F) did not show a reduction in body weight when compared with group II.

When compared to the standard control and all groups of animals, group I (50.35), group II (135.09), group III (67.61), group IV (114.01), group V (108.78), group VI (108.34) and group VII (69.14) had the highest percentage gain in body weight. On treatment with varying doses of standard medications and fractions, group III (67.48), group IV (21.08), group V (26.31), group VI (26.75), and group VII (65.95) lost the most percent body weight, indicating that HFD animals gained weight (Table 5).

##### 3.5.2. Effect of Wt-F of *B. diffusa* Extract on Organs Fat Pad Weight in SD Rats

The organ fat pad weight of group II animals is higher (*p* < 0.01) than group I animals for the heart (2.34), kidney (1.59), liver (2.76), and uterine (2.80). Compared to group II animals, the organ fat pad weight of sibutramine (5 mg/kg) and high-fat food treated in group III animals was significantly reduced (*p* < 0.01) on the heart (1.77 ± 0.14), kidney (1.19 ± 0.09), liver (1.67 ± 0.13), and uterus (1.54 ± 0.08). The fat pad on the heart (1.76 ± 0.09), kidney (1.21 ± 0.08), liver (1.62 ± 0.07), and uterus (1.56 ± 0.11) of group VII animals treated with Wt-F (200 mg/kg) was considerably reduced (*p* < 0.01) compared to group II animals. The organ fat pad of group IV, V, and VI animals treated with Hx-F, Chl-F, and nBut-F (200 mg/kg) revealed no significant differences from group II animals (Table 6).

##### 3.5.3. Effect of Wt-F of *B. diffusa* Extract on Food Intake in SD Rats

Table 7 shows the food intake pattern of HFD-fed and the normal group I animals. After seven days, HFD group II’s food consumption increased from 10.33 ± 1.80 to 14.83 ± 3.61. The food intake of group III rats treated with sibutramine (5 mg/kg) was 11.50 ± 2.39, which was substantially lower (*p* < 0.01) than group II. The food intake of group VII animals treated with Wt-F (200 mg/kg) was substantially lower (*p* < 0.01) than group II animals treated with Hx-F, Chl-F, and nBut-F (200 mg/kg).

##### 3.5.4. Effects of Wt-F of *B. diffusa* Extraction Lipid Profile in SD Rats

**Figure 9:**
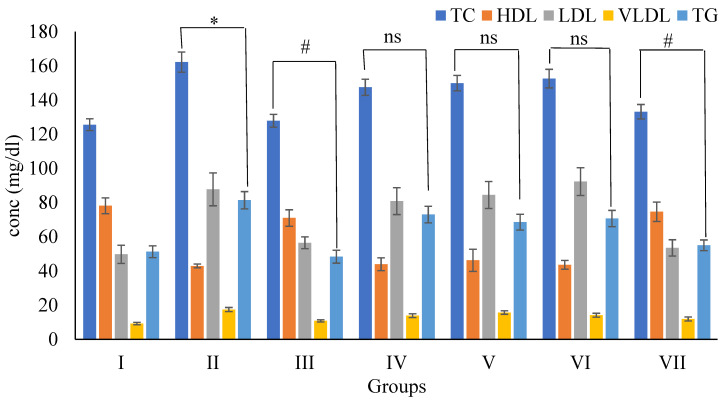
Effects of Wt-F of *B. diffusa* extract on lipid profile in SD rats after 60 days of treatment. Where TC = Total cholesterol; HDL = High density lipoprotein; LDL = Low density lipoprotein; VLDL = Very low-density lipoprotein; TG = Triglyceride. Data are expressed as Mean ± SEM of 6 rats in each group and *p*-value = * < 0.01 showed significance compared with respective group I, *p*-value = ^#^ < 0.01 showed significance compared respective with group II and ns = non-significant compared with respective group II.

Figure 9 shows that TC (162.17 ± 5.92 mg/dL), LDL (87.74 ± 9.59 mg/dL), VLDL (17.47 ± 1.19 mg/dL), and TG (81.41 ± 5.04 mg/dL) level was significantly elevated (*p* < 0.01). In contrast, HDL (42.94 ± 1.12) levels were lower (*p* < 0.01) in group II than in group I. Compared to group II animals, sibutramine (5 mg/kg) treated group III animals had lower TC (127.89 ± 3.75 mg/dL), LDL (56.49 ± 3.45 mg/dL), VLDL (10.84 ± 0.67 mg/dL), and TG (48.39 ± 3.77 mg/dL) levels, while HDL (71.01 ± 4.81 mg/dL) level was significantly increase. Wt-F treated group VII (200 mg/kg) animals had lower TC (133.17 ± 4.23 mg/dL), LDL (53.53 ± 4.75 mg/dL), VLDL (11.97 ± 1.18 mg/dL), and TG (55.02 ± 3.14 mg/dL) levels and higher HDL (74.64 ± 5.69 mg/dL) levels than group II. However, compared to group II animals, Hx-F, Chl-F, and n-But-F treatment (200 mg/kg) had no effect on lipid profile (TC, LDL, VLDL, TG, and HDL).

##### 3.5.5. Effects of Wt-F of *B. diffusa* Extraction AST & ALT Levels in SD Rats

**Figure 10:**
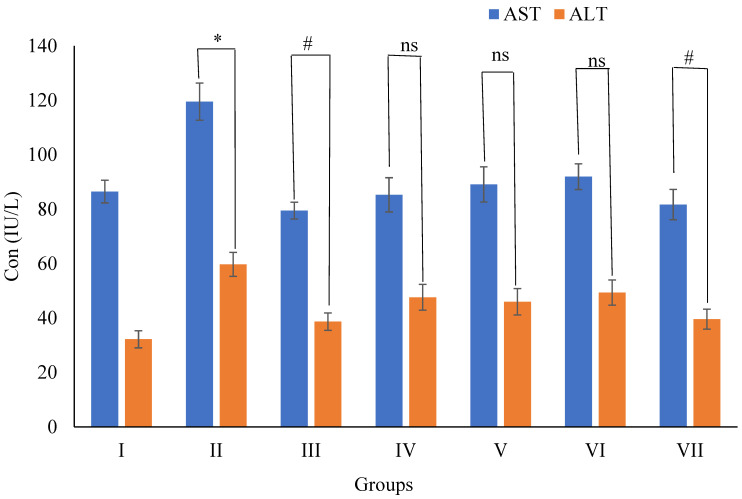
Effects of Wt-F of *B. diffusa* extract on AST and ALT levels in SD rats after 60 days of treatment. Where AST= Aspartate aminotransferase; ALT = Alanin pyrophosphate. Data are expressed as Mean ± SEM of 6 rats in each group. *p*-value = * < 0.01 showed significance compared with respective group I, *p*-value = ^#^ < 0.01 showed significance compared with respective group II and ^ns^ = non-significant compared with respective group II.

The AST and ALT (119.52 ± 6.84 and 59.72 ± 4.41 U/L) levels of group II animals were found to be higher (*p* < 0.01) than normal group I animals. The AST and ALT levels of group III animals treated with sibutramine (5 mg/kg) were substantially lower (*p* < 0.01) than group II animals. The treatment with Wt-F (200 mg/kg) reduced AST and ALT (91.97 ± 4.73 & 39.61 ± 3.66 U/L) significantly (*p* < 0.01) compared to group II, whereas the other fractions (200 mg/kg) like Hx-F, Chl-F, and n-But-F did not. When compared to group II animals, the levels of AST (85.29) and ALT (476.46, 45.98, 49.36 U/L) were non-significant (*p* > 0.05) (Figure 10).

##### 3.5.6. Effects of Wt-f of *B. diffusa* Extract on BUN and Creatinine Levels in SD Rats

**Figure 11:**
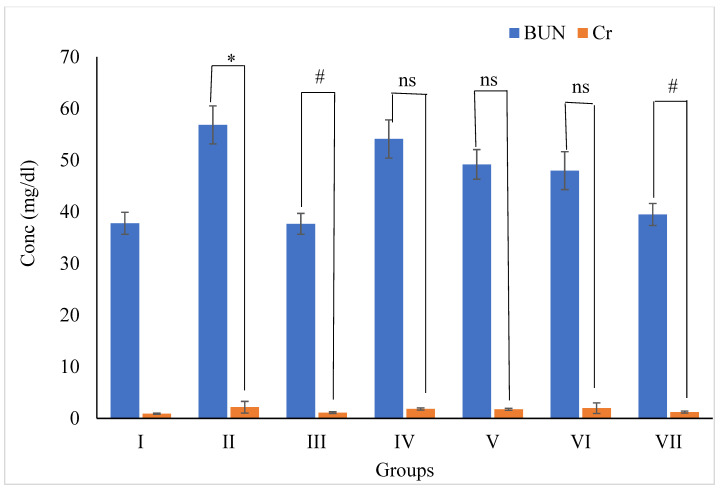
Effects of Wt-F of *B. diffusa* extract on BUN and creatinine in SD rats after 60 days of treatment. Where BUN = Blood Urea Nitrogen. Data are expressed as Mean ± SEM of 6 rats in each group. *p*-value = * < 0.01 showed significance compared with respective group I, *p*-value = ^#^ < 0.01 showed significance compared with respective group II and ^ns^ = non-significant compared with respective group II.

The BUN (56.84 ± 3.69 mg/dL) and creatinine (2.16 ± 1.13 mg/dL) concentrations of group II were considerably elevated (*p* < 0.01) as compared with group I as shown in Figure 11. The treatment of sibutramine (5 mg/kg) combined with a high-fat diet served to group III rats, resulted in BUN (37.67 ± 2.03 mg/dL) and serum creatinine (1.11 ± 0.17 mg/dL) being considerably reduced (*p* < 0.01) compared with group II animals. With the administration of Wt-F at a dose of 200 mg/kg daily combined with a high-fat meal in group VII, the BUN (39.49 ± 2.14 mg/dL) and creatinine (1.21 ± 0.19 mg/dL) levels significantly (*p* < 0.01) decreased when compared with group II. In contrast, in the other fractions such as Hx-F, Chl-F, and n-But-F administered at 200 mg/kg, the BUN and creatinine levels did not demonstrate significant (*p* > 0.05) effects compared with group II.

## 4. Discussion

With autodock 4.2, punarnavine, boeravinone B, and eupalitin were docked onto the active site of the cannabinoid receptor, with punarnavine, boeravinone B, and eupalitin binding to cannabinoid receptors quite well. The co-crystallized ligand sibutramine demonstrated equivalent binding to the crystal structure. These compounds’ interactions with cannabinoid receptors were extremely similar in their bound states. Leu57A, Tyr59A, Gly121A, Tyr151A, Tyr59B, Tyr119B, and Gly121B were involved in hydrophobic interactions with punarnavine, boeravinone B, and eupalitin [35]. Thus, the result of this study shows that by blocking the CR1 we can get the beneficial effect of the constituents (punarnavine, boeravinone B, and eupalitin) as a potent anti-obesity agent.

High throughput screening is one of the best options for fast screening of natural chemicals, in silico ADME studies, and molecular docking. In pharmacology, these computer-aided techniques help explain or predict toxicological consequences. The drug development process is arduous and involves significant investments and undertakings at every level of bioactive drug discovery which can lead to massive losses for a company. Notably, the ADME investigation revealed accurate information about the selected compounds’ pharmacokinetic and toxicity profiles, which will aid future therapeutic development [36].

The impact of *B. diffusa* root fractions on CB receptor blockage in obese SD rats is described here. The compound’s ability to lower food consumption in this animal was expected, as it has been evidenced in earlier investigations in rodents and primates [37,38,39]. We found the same in our findings. Rats were fed a high-fat diet with concurrent medication treatment [40]. Comprehensive strategies that include lifestyle changes and therapeutic interventions are required. Restoring a healthy energy balance requires both exercise and diet. Caloric restriction of a balanced, nutrient-dense diet is strongly recommended to prevent obesity growth [41].

Several researchers suggested that the consumption of calorically dense high-fat diets increased body weight in the HFD-fed untreated group of rats relative to the normal control group throughout the experimental period. The same results were observed in our study, i.e., with a high-fat diet, the Wt-F of *B. diffusa* extract (200 mg/kg/d) significantly lowered body weight (*p* < 0.01) compared to group II animals. While Hx-F, Chl-F, and n-But-F did not influence body weight (*p* > 0.05).

When the Wt-F of *B. diffusa* extract (200 mg/kg/d) was given in combination with a high-fat meal, the relative fat pad weight was considerably reduced (*p* < 0.01) compared to group II animals. Other fractions, such as Hx-F, Chl-F, and n-But-F, did not influence relative fat pad weight (*p* > 0.05). One of the key mechanisms for preventing fat storage is to promote lipolysis in adipocytes [42]. Furthermore, there have been numerous findings demonstrating regional changes in adipose metabolism, including nutritional therapy or exercise responsiveness, as well as lipolysis-promoting hormones [43].

Obesity prevention and treatment require both sides to fit, to prevent the rebound effect and subsequent weight gain [44]. Anandamide and 2-arachidonoylglycerol are two examples of endocannabinoid anabolic and catabolic enzymes [45]. The endocannabinoid is involved in both cerebral and distal energy metabolism [46]. Endocannabinoids act as retrograde synaptic plasticity neuromodulators of appetite and food intake, lipogenesis, and cravings [47,48]. Local endocannabinoids prevent fat accumulation. Thus, endocannabinoid blockade may cause calorie-independent lipolysis [49] and helps in obesity and metabolic illnesses [50]. Its intricacy has lately been confirmed by the discovery of numerous mediators that are biochemically linked to endocannabinoids and their neurotransmitters. Based on the above data, we believe our unique combination of lifestyle and pharmaceutical therapy could help cure obesity and its related cardio-renal disorders, which effectively blocks CB1 receptors and inhibits food intake, affecting body weight and lipid profiles, and parameters related to cardiovascular and renal health [51].

Obesity is connected to the over-activation of the endocannabinoid system, cannabinoid receptors (CB1 and CB2) and endogenous ligands (endocannabinoids). Caspase-1 expression is high in the brain but low in other tissues. Caffeine-binding receptor 1 (CB1) activating CB1 stimulates hunger and may cause obesity [52].

With a high-fat diet, the Wt-F of *B. diffusa* extract (200 mg/kg) significantly lowered TC, HDL, VLDL, TG, and LDL levels (*p* < 0.01) compared to group II animals. Other fractions including Hx-F, Chl-F, and n-But-F do not influence lipid profile (*p* > 0.05). The phytoconstituent -sitosterol is structurally similar to cholesterol and has been shown to lower cholesterol levels in plasma [53].

HFD supplementation increased blood TG, TC, and LDL-C while lowering HDL-C [42]. High TC, TG, and LDL values raise CVD risk [54]. The stimulation of gastric lipases, intestinal fat absorption, and lipolysis may affect the lipid profile. Impaired insulin activity is linked to lipid overload. Reduced hepatic and muscle glucose uptake resulted in hyperlipidemia due to increased adipose fat mobilization and insulin resistance [55]. Adding a HFD to rats’ plasma improved their lipid profile.

Obesity raises liver weight due to improper glycosylation or fibrosis, resulting in lipid accumulation in the cytoplasm, and increased serum AST and ALT levels [56]. The improvement in these irregular deviations mirrors the improvement in hepatopathies [57].

ALT and AST are two classic liver enzymes. They are enzymes that have been used to measure hepatotoxicity [58], particularly ALT, which is present in the cytoplasm of the rat liver. The two enzymes’ activity in the blood has been shown to rise in hazardous environments [59]. For example, ALT has been linked to hepatic fat deposition and insulin resistance, both of which contribute to metabolic syndrome [60]. The HFD obese groups’ ALT and AST activities were considerably (*p* < 0.01) higher than the normal control group’s [61]. Within the HFD groups, the water fraction significantly (*p* < 0.01) lowered ALT and AST while the other fractions (Hx-F, Chl-F, and But-F) did not.

Obesity has also been linked to renal impairment [62]. Obesity induces antioxidant system impairment in SD rats by increasing lipid peroxidation and decreasing GSH in the kidneys [63]. In the present study, obese rats showed a significant increase in serum creatinine and blood urea nitrogen levels compared with normal group animals. The water fraction had lower creatinine and BUN levels (*p* < 0.1), but not the other fractions (*p* > 0.05). Increased creatinine and BUN levels imply reduced pore shrinkage due to renal tubule cell proliferation and fibrosis [64].

## 5. Conclusions

The focus of this research was to find out the amazing therapeutic potential of *B. diffusa.* It is undeniable that the anti-obesity activity of *B. diffusa* is attributed because of several phytochemical elements in it. Overall, endocannabinoids appear to be an important part of the systems that regulate food and body weight, and they are orexigenic. Our experiments also show that drinking water fraction of *B. diffusa* blocks the cannabinoid receptor and thus has a clear anti-obesity impact in high-fat diet obese animals. Obese rats lost weight and adiposity by drinking water fraction of the plant extract. Nonetheless, understanding the pharmacological and therapeutic potential in the scientific environment will require more than just understanding the probable mechanism behind the anti-obesity activity. It will be a topic of significant interest and focus for future research.

## Figures and Tables

**Table 1:** Rf values and area of each spot of HPTLC fingerprinting of methanolic extract.

S. No.	Extract	λ (nm)	Rf Values	Area
1 2	***B. diffusa***	254	0.11, 0.28 0.38, 0.43 0.61, 0.71 0.90, 0.96	625.52, 4821.79 635.28, 498.76 4338.61, 4487.26 4651.92, 4367.64
		366	0.61, 0.90	6647.29, 1029.34

**Table 2:** Absorption, distribution, metabolism, excretion, and toxicity (ADMET) analyses of the selected bioactive compounds.

Molecule	Eupalitin	Boeravinone B	Punarnavine
Formula	C17H14O7	C17H12O6	C18H15NO4
Molecular weight	330.29 g/mol	312.27 g/mol	309.32 g/mol
No. of heavy atoms	24	23	23
No. Aromatic atoms	16	16	16
Fraction Csp3	0.12	0.12	0.17
No. rotatable bonds	3	0	2
No. H-bond acceptors	7	6	4
No. H-bond donors	3	3	0
Mol. Refractivity	86.97	82.63	87.46
TPSA	109.36 Å^2^	100.13Å^2^	49.69 Å^2^
iLOGP	2.27	2.47	3.14
XLOGP	2.82	2.39	3.12
WLOGP	2.59	2.24	2.94
MLOGP	0.07	0.87	1.65
SILICOS -IT Log P	2.59	2.62	3.47
Log *P*	2.59	2.12	2.87
ESOL Log S	−3.96	−3.80	−4.11
Solubility (mg/mL)	3.62 × 10^−^^2^	4.99 × 10^−2^	2.42 × 10^−2^
Solubility (mol/L)	1.10 × 10^−4^	1.60 × 10^−4^	7.83 × 10^−5^
Class	Soluble	Soluble	Moderately soluble
Log S (Ali clss)	−4.77	−4.13	−3.83
Solubility (mg/mL)	5.56 × 10^−3^	2.29 × 10^−2^	4.55 × 10^−2^
Solubility (mol/L)	1.68 × 10^−5^	7.34 × 10^−5^	1.47 × 10^−4^
Class	Moderately soluble	Moderately Soluble	Soluble
SILICOS-IT	−4.63	−4.56	−5.55
Silicos-IT Class	Moderately soluble	Moderately soluble	Moderately soluble

**Table 3:** Pharmacokinetics profile of the selected bioactive compounds.

Molecule	Eupalitin	Boeravinone B	Punarnavine
GI absorption	High	High	High
BBB permeant	No	NO	Yes
Pgp substrate	No	NO	No
CYP1A2 inhibitor	Yes	Yes	Yes
CYP2C19 inhibitor	No inhibitor	No inhibitor	No inhibitor
CYP2C9 inhibitor	Yes	No	Yes
CYP2D6 inhibitor	Yes	Yes	Yes
CYP3A4 inhibitor	Yes	Yes	Yes
Log *K*_p_ (skin permeation) (cm/s)	−6.31	−6.51	−5.97
Lipinski No of violation	0	0	0
GHOS	Yes	Yes	Yes
Veber	Yes	Yes	Yes
EGAN No. violation	Yes	Yes	Yes
MUEGGE No violation	Yes	Yes	Yes
Bioavailability Score	0.55	0.55	0.55
PAINS	0	0	0
BRENK	0	0	0
Lead likeness	Yes	Yes	Yes
Synthetic accessibility	3.40	3.82	2.78

**Table 4:** The molecular docking revealed the possible binding mechanisms of Cannabinoid receptors with the selected compounds.

Target	Ligands Chemical Structure	Binding Energy Score (Kcal/Mol)	H-Bond Interaction	Hydrophobic Interaction	H-Bond Distance
Cannabinoid Receptor	Sibutramine	+61.22	Thr105, Tyr106, Lys107, Arg112, Asn235, Ala324, Glu325, Cys326	ARGA:112: THR A:105 GLU A:325, ASN A:235:O	3.20207, 3.08945
	Boeravinone B	−3.11	Thr105, Tyr106, Lys107, Arg112, Asn235, Glu325 Glu377	THR A: 105, GLY A:329, TYR A:106, LEU A:113, LYS A:107, GLU A:377, LYSA:107, ARG A:112, THR A:105, ARG A:11, THR A:105, ASN A:235, GLU A:325, ASN A:235	2.73705, 3.01712 2.62171, 2.22553 2.98739, 3.20207 2.44576, 3.08945
	Eupalitin	−4.28 kcal	Thr105, Tyr106, Lys107, Arg112 Glu234, Asn235, Ala324, Glu325 Cys326, Glu377	LYS A:107, GLU A:377, ARG A:112, THR A:105, ARG A:112, GLU A:325, ASN A:235:O	2.62171, 3.20207 2.66729, 3.08945
	Punarnavine	−0.91	Arg96, Tyr106, Lys107, Tyr108 Leu109, Asn110, His231, Asp233, Glu234, Asn235, Arg322	A:ARG96:, A:ASN 205, A:TYR 106, GLU A:234, LYS A:107, GLU A:377,TYR A:108, THR A:111, GLU A:234, ARG A:322	2.2459, 2.33441 2.44846, 2.60911 2.62171, 2.63316

**Table 5:** Effects of Wt-F of *B. diffusa* extract on body weight in SD rats after 60 days of treatment.

Groups	Treatments	Initial Body Weight (g)	Final Body Weight (g)	% Increase in Body Weight	% Decrease in Body Weight
I	Normal Control (NC)	135.30 ± 3.27	203.42 ± 2.24	50.35	-
II	High Fat Diet (HFD)	136.12 ± 2.47	320.00 ± 5.01 *	135.09	-
III	HFD + Sibutramine (5 mg/kg)	135.23 ± 2.39	226.67 ± 2.01 ^#^	67.61	67.48
IV	HFD + Hx-F (200 mg/kg)	137.50 ± 1.71	294.27 ± 4.24 ^ns^	114.01	21.08
V	HFD + chlo-F (200 mg/kg)	137.50 ± 3.35	287.08 ± 4.67 ^ns^	108.78	26.31
VI	HFD + n-but-F (200 mg/kg)	136.40 ± 1.54	284.17 ± 3.21 ^ns^	108.34	26.75
VII	HFD + wt-F (200 mg/kg)	135.23 ± 2.38	228.73 ± 4.17 ^#^	69.14	65.95

Data are expressed as Mean ± SEM of 6 rats in each group. *p*-value = * < 0.01 showed significance compared with respective group I; *p*-value = ^#^ < 0.01 showed significance compared with respective group II and ^ns^ = non-significant compared with respective group I.

**Table 6:** Effects of *Wt-F of B. diffusa extract* on the visceral fat pad in SD rats after 60 days of treatments.

Groups	Treatments	Heart (g)	Kidney (g)	Liver (g)	Uterus (g)
I	Normal Control (NC)	1.75 ± 0.11	1.17 ± 0.09	1.54 ± 0.09	1.49 ± 0.07
II	High Fat Diet (HFD)	2.34 ± 0.14 *	1.59 ± 0.18 *	2.76 ± 0.16 *	2.80 ± 0.06 *
III	HFD + Sibutramine (5 mg/kg)	1.77 ± 0.14 ^#^	1.19 ± 0.09 ^#^	1.67 ± 0.13 ^#^	1.54 ± 0.08 ^#^
IV	HFD + Hx-F (200 mg/kg)	2.22 ± 0.17 ^ns^	1.28 ± 0.11 ^ns^	2.61± 0.17 ^ns^	2.15 ± 0.10 ^ns^
V	HFD + chlo-F (200 mg/kg)	2.12 ± 0.20 ^ns^	1.31 ± 0.12 ^ns^	2.49 ± 0.18 ^ns^	2.11± 0.11 ^ns^
VI	HFD + n-but-F (200 mg/kg)	2.29 ± 0.12 ^ns^	1.29 ± 0.04 ^ns^	2.71 ± 0.10 ^ns^	2.09 ± 0.13 ^ns^
VII	HFD + wt-F (200 mg/kg)	1.76 ± 0.09 ^#^	1.21 ± 0.08 ^#^	1.62 ± 0.07 ^#^	1.56 ± 0.11 ^#^

Data are expressed as Mean ± SEM of 6 rats in each group. *p*-value = * < 0.01 showed significance compared with respective group I, *p*-value = ^#^ < 0.01 showed significance compared with respective group II and ^ns^ = non-significant compared with respective group II.

**Table 7:** Effects of *Boerhavia diffusa* root fractions (WT-F) on food intake in SD rats after seven days of treatment.

Groups	Treatments	Initial Day (g) 0 Day	The Final Day (g) 7th Day
I	Normal Control (NC)	9.67 ± 1.23	10.33 ± 1.80
II	High Fat Diet (HFD)	8.17 ± 1.35	14.83 ± 3.61 *
III	HFD + Sibutramine (5 mg/kg)	10.83 ± 2.12	11.50 ± 2.39 ^#^
IV	HFD + Hx-F (200 mg/kg)	8.01 ± 1.29	13.67 ± 2.55 ^ns^
V	HFD + chlo-F (200 mg/kg)	10.33 ± 1.43	13.17 ± 3.27 ^ns^
VI	HFD + n-but-F (200 mg/kg)	9.33 ± 1.28	13.33 ± 3.05 ^ns^
VII	HFD + wt-F (200 mg/kg)	9.67 ± 1.75	11.50 ± 2.43 ^#^

Data are expressed as Mean ± SEM of 6 rats in each group. *p*-value = * < 0.01 showed significance compared with respective group I, *p*-value = ^#^ < 0.01 showed significance compared with respective group II and ^ns^ = non-significant compared with respective group I.

## Data Availability

Not applicable.

## Abbreviations

2-AG: 2-arachidonoyl glycerol; ADME: Absorption, distribution, metabolism, and excretion; ALT: Alanine aminotransferase; ANOVA: Analysis of variance; AST: Aspartate aminotransferase; b.w.: Bodyweight; BMI: Body mass index; BUN: Blood urea nitrogen; But-F: Butanol fraction of *Boerhavia diffusa*; CBI: Cannabinoid; Chl-F: Chloroform fraction of *Boerhavia diffusa*; CPCSEA: Committee for the Purpose of Control and Supervision of Experiments on Animals; CR: Cannabinoid receptor; CVD: Cardiovascular disease; FDA: Food drug administration; GPF: Grid parameter file; GLG: Grid log file; DLG: Docking log file; GROMACS: Groningen Machine for Chemical Simulations; GSH: Glutathione; HB: Hydrogen bond; HDL: High-density lipoprotein; HDL: High-density lipoprotein; HFD: High-fat diet; HIA: Human intestinal absorption; HPTLC: High-performance thin-layer chromatography; Hx-F: Hexane fraction of *Boerhavia diffusa;* Kg: kilogram; LDL: Low-density lipoprotein; LINCS: Linear Constraint Solver; MD: Molecular Docking; 5HT: 5-hydroxytryptamine; NPT: Nuclear Non-Proliferation Treaty; NVT: Network Virtual Terminal; OPLA-AA: Optimized Potentials for Liquid Simulations-All Atom; PDBQT: Protein Data Bank Partial Charge (Q) & Atom Type; Rf: Retention factor; *Rg*: *Radius* of gyration; RMSD: Root Mean Square Deviation; RMSF: Root mean square fluctuation; SD: Sprague–Dawley; TC: Total cholesterol; TG: Triglyceride; TIP3P: transferable intermolecular potential with 3 points; TLC: thin-layer chromatography; TPSA: Topological surface area; VLDL: Very low-density lipoprotein; WHO: World Health Organization; Wt-F: Water fraction of *Boerhavia diffusa*.
